# Patient experience of lasting negative effects of psychological interventions for anxiety and depression in secondary mental health care services: a national cross-sectional study

**DOI:** 10.1186/s12888-021-03588-2

**Published:** 2021-11-17

**Authors:** Aisling McQuaid, Rahil Sanatinia, Lorna Farquharson, Prisha Shah, Alan Quirk, David S. Baldwin, Mike Crawford

**Affiliations:** 1 Division of Psychiatry, Imperial College London, London, UK; 2 School of Psychology, University of East London, London, UK; 3 NCAAD Service User Advisor, Royal College of Psychiatrists, London, UK; 4 CCQI Head of Clinical Audit and Research, Royal College of Psychiatrists, London, UK; 5 Psychiatry, Clinical and Experimental Sciences, Faculty of Medicine, University of Southampton, Southampton, UK

## Abstract

**Background:**

Patients who undergo psychological treatment can report both negative and positive effects, but evidence of factors influencing the likelihood of negative effects is limited.

**Aims:**

To identify aspects of the organisation and delivery of secondary care psychological treatment services that are associated with patient experiences of negative effects.

**Method:**

Cross-sectional survey of people with anxiety and depression who ended psychological treatment delivered by 50 NHS trusts in England. Respondents were asked about how their treatment was organised and delivered and whether they experienced lasting negative effects.

**Results:**

Of 662 respondents, 90 (14.1%) reported experiencing lasting negative effects. People over the age of 65 were less likely than younger respondents to report negative effects. There was an association between reporting neutral or negative effects and not being referred at what respondents considered to be the right time (OR = 1.712, 95% CI = 1.078–2.726), not receiving the right number of sessions (OR = 3.105, 95% CI = 1.934–4.987), and not discussing progress with their therapist (OR 2.063, 95% CI = 1.290–3.301).

**Conclusions:**

One in seven patients who took part in this survey reported lasting negative effects from psychological treatment. Steps should be taken to prepare people for the potential for negative experiences of treatment, and progress reviewed during therapy in an effort to identify and prevent negative effects.

## Introduction

One in six adults experience a common mental health problem such as anxiety and depression [1]. People with anxiety and depression experience emotional distress, reduced quality of life [2], and impaired social functioning [3]. The National Institute for Health and Clinical Excellence in England recommend psychological interventions as first line treatments for anxiety and depression [4, 5]. In England, most people who undergo psychological treatment for anxiety and depression receive it in primary care. However, patients with more severe conditions, those with complex difficulties associated with coexisting conditions, and people judged at high risk of suicide are treated by specialist teams based in secondary care mental health services.

Although psychological interventions for depression and anxiety generally have positive effects [6, 7], less consideration has been given to their potential for negative ones [8, 9]. Outcome measures are often used to monitor for adverse effects of treatment [10], however the results of these measures do not take into account patients’ subjective experiences of therapy. Lasting negative effects may include a worsening of symptoms, or the development of new ones, such as anger, loss of self-esteem and anxiety following treatment [11].

Secondary analysis of data from a national survey of 14,587 patients receiving treatment in England found that 5% of patients reported lasting negative effects from the psychological treatment they had received [11]. With a high potential for lasting negative effects, it is important to examine their aetiology in order to prevent them [12].

The likelihood of negative effects may be greater among people with personality disorder and people who have had childhood trauma, which are commonly seen in secondary care and frequently associated with adverse events of therapy [13–16]. Other patient factors which are associated with negative effects include unemployment, disability, and expectations of treatment benefits [17]. Age, ethnicity, and sexuality are also associated with the likelihood of patient-reported negative effects: those over 65 years are less likely than younger patients to report negative outcomes but people from sexual and ethnic minorities are more likely to report such concerns [11]. Negative effects may also be more common among patients who are less optimistic about their treatment [18] and among those who do not feel they receive enough information about their treatment before it begins [11]. If people have preferences about their treatment, which are not met, they are more likely to report a poor outcome of therapy [19]. Although patient demographic and clinical factors appear to influence the likelihood of negative experience of psychological treatment, less is known about the relationship between the process of delivering psychological treatment and reports of negative outcomes.

Service structures, policies, and constraints may influence whether patients experience negative effects of their treatment. A thematic analysis of factors which lead to patient reported negative experiences included experiences not matching expectations, and services providing little or no information about what the service offered [20]. Data from the Improving Access to Psychological Treatment (IAPT) programme, show that better clinical outcomes were achieved by services that offered more treatment sessions to patients, and by services that followed NICE treatment recommendations more closely [21]. Delay in referral to appropriate psychological services may also have a negative impact on psychotherapy outcomes [22]. However, gaps remain in our understanding of the contribution that the process of delivering psychological treatment has on the likelihood of patients experiencing negative effects.

We therefore undertook a secondary analysis of data from a survey of 662 patients conducted as part of the National Clinical Audit of Anxiety and Depression [23]. The survey was run by the Royal College of Psychiatrists (RCPsych) in collaboration with the British Psychological Society (BPS). The audit involved an examination of process and outcomes of care using data from case-notes, a survey of therapists and a patient survey. The main analysis for this report uses data from the patient survey. The aim of the analysis was to identify aspects of the organisation and delivery of secondary care psychological therapy services that are associated with reported negative effects. We aimed to build on our previous study by investigating the prevalence of negative effects among people treated by secondary care services rather than those treated predominately by IAPT, in a sample of patients who had completed their treatment. We also wanted to examine the effects of new predictor variables, including whether patients had had discussions previous experience of therapy with their therapist and whether they discussed their progress during their therapy sessions.

## Method

All 54 providers of NHS secondary care mental health services (known as ‘Trusts’) in England were invited to take part in the case-note audit, and 50 (92.6%) submitted data. Each Trust was asked to register all teams that deliver psychological treatment to adults (aged 18 or over) with anxiety and depression, including both inpatient and community services.

All psychological therapy teams were asked to compile a list of all patients who ended psychological therapy between 1st September 2017 and 31st August 2018. This included people who completed their treatment, as well as those who did not complete or dropped out of treatment. The audit team at the Royal College of Psychiatrists then randomly selected 30 patients from each team who were sent a copy of the survey. Any patients with a coexisting diagnosis of a psychotic condition were excluded from the survey. If fewer than 30 patients had completed treatment during this 12-month period, all eligible patients were sent a copy of the survey.

The National Research Ethics Service and the Ethics and Confidentiality Committee of the National Information Governance Board advised that formal ethical approval was not required because this was an audit and patient identifiable data were not collected. All procedures contributing to this work comply with the ethical standards of the relevant national and institutional committees on human experimentation and with the Helsinki Declaration of 1975, as revised in 2008. Approval for the secondary analysis of the data in this study was obtained from the Healthcare Quality Improvement Partnership prior to the start of data analysis.

One of the questions in the audit asked service users to indicate whether they agreed or disagreed with the statement: “*I have experienced lasting bad effects from the treatment*”, to which they could respond “strongly agree”, “slightly agree”, “neutral”, “slightly disagree” or “strongly disagree”.

Potential predictor variables were chosen following a review of previous literature and discussion with a service user advisor to the project (P.S.): they included demographic factors (age, gender, ethnicity), along with the following aspects of the organisation and delivery of care where patients could respond “strongly agree”, “slightly agree”, “neutral”, “slightly disagree” or strongly disagree”:

- I was referred to therapy at the right time
- The waiting time for my treatment to start was reasonable
- I received enough information about my therapy before it began
- I received the right number of therapy sessions from this service
- My therapist and I agreed goal(s) for my therapy
- I had a discussion with my therapist about my previous therapy and experiences
- I had a discussion with my therapist about my overall care (e.g., medication, appointments with your care coordinator)
- Did you and your therapist discuss progress during your therapy?

### Analysis

The primary outcome for this study was a service user report of ‘lasting bad effects from treatment’. A preliminary analysis of data using crosstabulation indicated that the demographic characteristics (age, gender, ethnicity) of service users who stated that they were ‘neutral’ about whether they had experienced a lasting bad effect from their treatment were more closely aligned to those reporting negative effects than positive effects, we therefore included the responses of those who were neutral about having experienced lasting bad effects with those who reported that they had such experiences. We used the same approach to convert other items to dichotomous variables prior to the main analysis. A univariate analysis was undertaken to explore associations between the primary outcome and demographic factors (age, gender, ethnicity), and predictor variables on the process of care.

We examined whether there was clustering between variables at the level of service but found no evidence of clustering (*p* = 0.146, 95% CI = 0.051–0.753). We generated a multivariate model of factors which predicted the likelihood of negative effects using binary logistic regression. We used backwards elimination to delete any variables from the model whose removal did not result in any statistically significant loss of fit. We also used data from the case-note audit to explore the representativeness of those who completed the survey compared to those who did not. Analysis was completed using the statistical package IBM SPSS (version 26).

## Results

Surveys were sent to 4462 service users treated by 232 psychological therapy services across the country: 662 surveys (14.8, 95% CI 13.82–15.92) were completed and included in this analysis.

The characteristics of the service-users who completed the survey are presented in Table 1 along with the aggregate data from the case-note audit. Survey respondents were more likely to be female, older and white compared with non-respondents. Overall, 14.1% (*n* = 90) of respondents reported experiencing lasting bad effects, of those 6.1% (*n* = 39) strongly agreed and 8% (*n* = 51) slightly agreed, 13.7% (*n* = 87) were neutral, and 72.2% (*n* = 459) indicated that they had not experienced lasting bad effects, with 7.7% (*n* = 49) who slightly disagreed and 64.5% (*n* = 410) strongly disagreed. When ‘agree’ and ‘neutral’ categories were combined into dichotomous variable, the total was 27.8% (*n* = 177). Univariate associations between the experience of lasting bad effects and predictor variables are presented in Table 2.

**Table 1:** Demographic characteristics of study participants

Demographic Characteristics	Study sample, n (%)	Sample included in the case-note audit	Difference in proportions (95% CI)
Age, years	624	4462	
18–25	38 *(6.1)*	680 *(15.2)*	−9.15 *(−6.67 to − 11.16)*
26–35	75 *(12)*	923 *(20.7)*	−8.67 (− 5.56 to − 11.36)
36–45	94 *(15.1)*	808 *(18.1)*	−3.04 *(− 0.25 to − 5.96)*
46–55	135 *(21.6)*	811 *(18.2)*	3.46 *(0.12 to 7.12)*
56–65	88 *(14.1)*	436 *(9.8)*	4.33 (*1.59 to 7.48)*
> 65	194 *(31.1)*	804 *(18)*	13.07 *(9.3 to 17.05)*
Gender	626	4459	
Male	208 *(33)*	1484 *(33.3)*	−0.21 *(−3.88 to 4.12)*
Female	418 (66.5)	2966 *(66.5)*	0.06 *(−3.86 to 4.16)*
Ethnicity	625	4202	
White	557 *(89.1)*	3691 *(87.8)*	1.28 *(−1.65 to 3.78)*
Mixed / Multiple	14 *(2.2)*	141 *(3.35)*	−1.12 *(−0.55 to − 2.25)*
Asian	29 *(4.6)*	181 *(4.3)*	0.33 *(−1.27 to 2.45)*
Black	17 *(2.7)*	94 (*2.2)*	0.48 *(−0.71 to 2.22)*
Other	8 *(1.3)*	95 *(2.3)*	−0.98 *(− 0.41 to − 1.83)*

**Table 2:** Univariate analysis of neutral or lasting bad effects – demographic factors and process of care

	Neutral or lasting negative effects, n/N (%)	OR (95% CI)
Age, years		
> 65	29/181 (16)	1
18–25	12/38 (31.6)	2.419 (1.097–5.335)
26–35	24/72 (33.3)	2.621 (1.395–4.924)
36–45	23/91 (25.3)	1.773 (0.956–3.287)
46–55	49/133 (36.8)	3.057 (1.798–5.119)
56–65	25/85 (29.4)	2.184 (1.183–4.030)
Gender		
Female	112/404 (27.7)	1
Male	48/198 (24.2)	0.834 (0.564–1.234)
Ethnicity		
White	138/535 (25.8)	1
Mixed	2/14 (14.3)	0.479 (0.106–2.169)
Asian	8/28 (28.6)	1.151 (0.496–2.672)
Black	5/16 (31.3)	1.308 (0.446–3.830)
Other	4/8 (50)	2.877 (0.710–11.659)
*I was referred to therapy at the right time*		
Agree	76/412 (18.4)	1
Neutral/Disagree	97/205 (47.3)	3.971 (2.742–5.751)
*The waiting time for my treatment to start was reasonable*		
Agree	67/357 (18.8)	1
Neutral/Disagree	101/250 (40.4)	2.934 (2.034–4.233)
*I received enough information about my therapy before it began*		
Agree	71/382 (18.6)	1
Neutral/Disagree	99/226 (43.8)	3.415 (2.363–4.934)
*I received the right number of therapy sessions from this service*		
Agree	64/377 (17)	1
Neutral/Disagree	113/252 (44.8)	3.976 (2.757–5.733)
*My therapist and I agreed goals for my therapy*		
Agree	103/478 (21.5)	1
Neutral/Disagree	73/147 (49.7)	3.592 (2.432–5.405)
*I had a discussion with my therapist about my previous therapy and experiences*		
Agree	116/462 (25.1)	1
Neutral/Disagree	60/162 (37)	1.755 (1.198–2.571)
*I had a discussion with my therapist about my overall care*		
Agree	90/423 (21.3)	1
Neutral/Disagree	84/199 (42.2)	2.703 (1.876–3.894)
*Did you and your therapist discuss your progress?*		
Yes	98/462 (21.2)	1
No/Unsure	79/166 (47.6)	3.373 (2.312–4.920)

The results of the multivariate analysis are presented in Table 3, which contains all the covariates that remained in the model after backwards regression*.* The likelihood of reporting neutral or lasting negative effects was greater among service users who felt they were not referred for therapy at right time, those who felt they did not receive the right number of sessions, and those who reported that they did not discuss their progress with their therapist.

**Table 3:** Multivariate analysis of factors associated with likelihood that patients reported neutral or lasting negative effects of treatment

Experience of treatment	OR (95%) CI	P
*Referred at right time*		
Agree	1	
Neutral/Disagree	1.712 (1.078–2.726)	0.023
*Received the right number of therapy sessions from this service*		
Agree	1	
Neutral/Disagree	3.105 (1.934–4.987)	0.000
*Discussed progress with therapist*		
Agree	1	
Neutral/Disagree	2.063 (1.290–3.301)	0.003

## Discussion

Analysis of data from 662 people who received psychological therapy for anxiety and depression in secondary care mental health services in England found that 14.1% reported a lasting negative effect as a result of their treatment, and 13.7% reported being ‘neutral’ about whether they had a negative effect. This is a higher proportion than the 5.2% of patients who reported negative effects in a previous investigation [11]. This is potentially due to the sample being derived exclusively from secondary care mental health services where the patients have more severe or complex difficulties. Patients over the age of 65 were less likely to report negative effects compared to younger people. This is consistent with previous findings [11].

The term ‘lasting’ was included in the patient survey following initial qualitative interviews prior to a previous round of this study, where it was acknowledged that patients may feel uneasy or unsettled during therapy, but that these difficult feelings were worked through during therapy. The term lasting was included as this implied that the negative effects last beyond the treatment. It is possible that this term is subjective and should be considered in any future investigation.

We found associations between the likelihood of experiencing neutral or lasting bad effects and three components of the organisation and delivery of services. First, there was an association between not being referred ‘at the right time’ for therapy and neutral or lasting bad effects. In a thematic analysis of 447 of the service-user surveys [24] this was identified as a major concern. Service users felt that therapy was not easily accessible and were concerned with difficulties meeting the threshold for services, with people being assessed as ‘too well’ to be referred for treatment. Our findings provide further evidence of the need to ensure that people with common mental health conditions have timely access to psychological treatment.

Not receiving ‘the right number’ of therapy sessions was also associated with experiencing neutral or lasting negative effects of treatment, mirroring the thematic analysis report which identified that the number of sessions received was often not enough to meet the service-user’s needs [24]. This is consistent with IAPT findings that services which offer a greater number of sessions to service-users have a more positive impact on recovery [21].

Not discussing progress with therapist showed the strongest relationship to likelihood of reporting neutral or lasting negative effects. This is of particular concern given previous reports that patients rarely disclose negative reactions to their therapists or services [20, 25].

In-session monitoring has been found to improve patient outcomes [26–28]. Discussions regarding patient progress may help maintain a therapeutic relationship, allow for collaboration, and modification of specific strategies for treatment [29]. A lack of discussion on progress may make it difficult for patients to engage in therapy [20]. Therapists may also have a differing view on patient progress [30]. These discussions may highlight patient’s experiences of therapy, identifying concerns patient may have and increasing the opportunities to avert negative outcomes. Despite the evidence that progress monitoring can be effective in improving treatment outcome, it remains an infrequent practice [31, 32].

There are a number of strengths of the present study. The data come from a large number of services across England. This study was closely based on a previous analysis of negative outcomes delivered mainly to patients in primary care [11]. However, unlike the previous study, all those who took part in this new study had completed their therapy when they completed the survey. Some patients have both positive and negative experiences during therapy, and working through difficult experiences can be part of the therapeutic process [33] Further investigation is required as to whether those who report lasting harm from their therapy also found it helpful.

Our survey questions were developed through in-depth interviews with patients and a person with lived experience of anxiety and depression helped to select items for inclusion in this analysis. While previous studies have examined patient factors and environmental factors that may increase the likelihood of negative effects of psychological treatments [17] we focussed on aspects of the organisation and delivery of services because these may be amenable to change.

Some important limitations must also be considered. The data are limited to service-user experiences in secondary care services. The response rate was low with 662 (14.8%) taking part out of the 4462 who were sent a copy: when compared to the data from the case-note audit, it was more likely for non-respondents to be younger, male, and from minority ethnic groups. We do not know if the proportion of those experiencing neutral or lasting bad effects of treatment would be the same among those who did not respond. It is also possible that responses to the survey questions may differ depending on whether the respondent was a completer or non-completer of therapy, however this information was not collected as part of the audit and so we were unable to include in our analysis. It would be useful for this to be considered for future studies. As neutral and agree categories were combined in order to calculate dichotomous variables, it is possible that some respondents were included who did not experience lasting adverse effects. This should be considered in future studies. The study design is reliant upon retrospective views of individual experiences rather than prospectively gathered routine service data. The cross-sectional design of the study means that we are not able to conclude that the associations we found are causal. It is possible that recall bias led people who may have negative experiences of therapy to indicate problems with when and how their therapy was delivered. Future prospective studies could reduce the potential for this bias by examining the process of care during therapy and subsequently assessing patient experiences of the outcome of their treatment.

Important factors that should be considered in future studies include clinician characteristics and the interactions between clinician and patient. It is not clear from this study the contribution these interactions may have upon a patient’s likelihood of reporting lasting negative effects from their treatment.

Our findings suggest that a significant proportion of service-users with anxiety and depression may report lasting negative effects of psychological treatment in secondary care: some people do not feel helped, but instead feel harmed. Services should inform service users prior to starting treatment that although most people who complete psychological treatment feel benefits, negative experiences are not infrequent [34]. Clinicians delivering psychological therapy in secondary care settings should ensure that they discuss progress with patients, and further efforts are needed to reduce waiting times and provide sufficient sessions for service-user’s needs to be addressed. Future research should aim to develop a better understanding the content of these lasting negative effects and how they affect clients to reduce and mitigate the risks. Longitudinal studies are needed to develop a better understanding of the relationship between the process and outcomes of psychological therapies. Experimental studies are needed to see if changing the way that therapy is delivered can help reduce the incidence of patient reported negative outcomes.

## Conclusion

One in seven patients receiving psychological therapy in secondary care mental health services for anxiety and depression reported experiencing lasting bad effects from their treatment. Being referred at the right time, receiving the right number of sessions, and discussing progress with the therapist may help reduce the likelihood of this adverse outcome.

## Data Availability

Applications for access to data from the National Clinical Audit of Anxiety and Depression should be made to the Healthcare Quality Improvement Partnership. Details of the process for obtaining these data are available at: https://www.hqip.org.uk/national-programmes/accessing-ncapop-data/#.YDPSHBP7Su5.
